# Post-Translational Modifications in HIV Infection: Novel Antiretroviral Strategies

**DOI:** 10.3390/cells15030243

**Published:** 2026-01-27

**Authors:** Yidong Sun, Siyi Yang, Youxi Ao, Wei Yu

**Affiliations:** 1 College of Life Sciences and Medicine, Zhejiang Sci-Tech University, Xiasha High-Tech Zone No. 2 Road, Hangzhou 310018, China; 2023332864030@mails.zstu.edu.cn (Y.S.); 2025010901003@mails.zstu.edu.cn (S.Y.); 2024332864084@mails.zstu.edu.cn (Y.A.); 2 Zhejiang Provincial Key Laboratory of Silkworm Bioreactor and Biomedicine, Hangzhou 310018, China

**Keywords:** human immunodeficiency virus, post-translational modification, therapeutic strategies

## Abstract

Human immunodeficiency virus (HIV) infection remains a major global health burden. Untreated HIV infection leads to CD4^+^ T-cell depletion and severe immune dysfunction, resulting in opportunistic infections, neoplastic changes, and death. Highly active antiretroviral therapy (HAART) is currently the standard treatment for HIV infection, but it cannot eliminate latent reservoirs. Post-translational modifications (PTMs) regulate protein trafficking, function, and degradation, and their in-depth investigation plays a crucial role in identifying novel biomarkers and therapeutic targets. PTMs exert a central regulatory role in HIV infection by both enhancing host restriction factors and contributing to latent infection. This dual role offers novel insights into potential therapeutic targets for activating latent viruses to make them visible to the immune system. This review highlights numerous PTMs associated with HIV infection, including acetylation, phosphorylation, palmitoylation, etc., and assesses their potential for curing HIV infection.

## 1. Introduction

Human immunodeficiency virus (HIV), a lentivirus belonging to the Retroviridae family [1], remains one of the world’s foremost health challenges. Since the onset of the HIV epidemic, over 40 million individuals have succumbed to acquired immunodeficiency syndrome (AIDS) [2]. The persistence of a latent viral reservoir, which remains undetectable by the immune system, renders complete eradication of HIV infection exceptionally difficult [3]. Current widely adopted antiretroviral therapy (ART) cannot eliminate these reservoirs, so that treatment cessation leads to viral rebound, hindering progress in HIV management [4,5].

**Figure 1:**
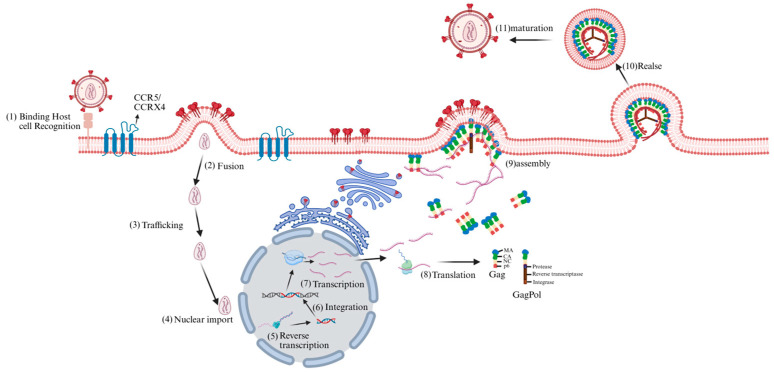
HIV life cycle. The HIV life cycle commences with viral interaction with host cell receptors (1), triggering fusion with the cell membrane and release of the viral core into the host cell cytoplasm (2). Subsequently, the core is transported through the cytoplasm (3), during which reverse transcription and nuclear import begin. At the nuclear pore complex, viral components are transported into the nucleus (4) and localized to transcriptionally active chromatin regions; concurrently, viral core uncoating and reverse transcription continue (5). Integration then occurs (6). Subsequently, viral genes are transcribed (7) and translated (8) into Gag polyprotein precursor, the GagPol polyprotein precursor, the viral envelope glycoproteins, and the regulatory and accessory viral proteins. Under the mediation of the Gag polyprotein, immature virus particles were assembled on the cell membrane (9) and released (10) outside the cell. Subsequently, the viral protease initiated a highly ordered cutting process, ultimately forming mature virus particles (11).

Based on genetic characteristics and viral antigenic differences, the virus is classified into the HIV-1 and HIV-2 subtypes [6], the former being characterized by higher virulence and broader distribution. As a canonical retrovirus belonging to the genus lentivirus, HIV irreversibly integrates into the host genome, features a prolonged clinical latency period, and possesses the ability to infect non-proliferating cells [7]. HIV primarily targets CD4^+^ T lymphocytes [8], and infection commences when the viral envelope protein gp120 binds to the host cell’s CD4 receptor, followed by association with CCR5 or CXCR4 as a co-receptor [9,10]. The fusion of the virus with the cell membrane is mediated by gp41, and once the viral core enters the cell, the RNA genome of the virus is reverse-transcribed into DNA. Thereafter, the viral DNA is integrated into the host genome by integrase, thereby forming a provirus [11,12,13,14]. When the latent HIV provirus is activated, the host RNA polymerase II initiates viral gene transcription, generating multiple viral transcripts, including full-length genomic RNA and various spliced RNAs, which are then transported to the cytoplasm. The viral RNA that enters the cytoplasm is subsequently translated into the Gag polyprotein precursor (also known as Pr55Gag), the GagPol polyprotein precursor, the viral envelope glycoproteins (Env glycoproteins), and the regulatory and accessory viral proteins [15]. The Gag protein relies on its matrix (MA) region to precisely target the plasma membrane as a viral assembly platform [16]. Subsequently, Gag recognizes the packaging signal of the 5 ′untranslated region of HIV RNA through its nucleocapsid (NC) region, recruits dimerized viral genomic RNA and drives Gag multimerization, forming immature viral particles that encapsulate viral RNA and GagPol [17]. At the same time, Env was incorporated into the viral envelope through the interaction between MA and the cytoplasmic tail of gp41. The newly released viral particles are still in an immature state [18]. Subsequently, the viral protease (PR) initiates a highly ordered cleavage cascade, cutting Gag and GagPol into MA, capsid (CA), NC, and multiple enzyme proteins, promoting the reassembly of CA into a conical mature capsid [19], completing the morphological remodeling of the virus and endowing it with full infectious ability (Figure 1).

In recent years, significant advances have been made in understanding the biomolecular mechanisms governing virus-host cell interactions. In particular, the characterization of post-translation modifications (PTMs) has shed light on how HIV modulates the cell cycle, transcription, translation, or the selective degradation of antiviral proteins [20]. Several studies have underscored the importance of PTMs in HIV infection, successfully employing modified enzyme inhibitors to reduce viral load or reactivate latent viruses [21,22].

**Figure 2:**
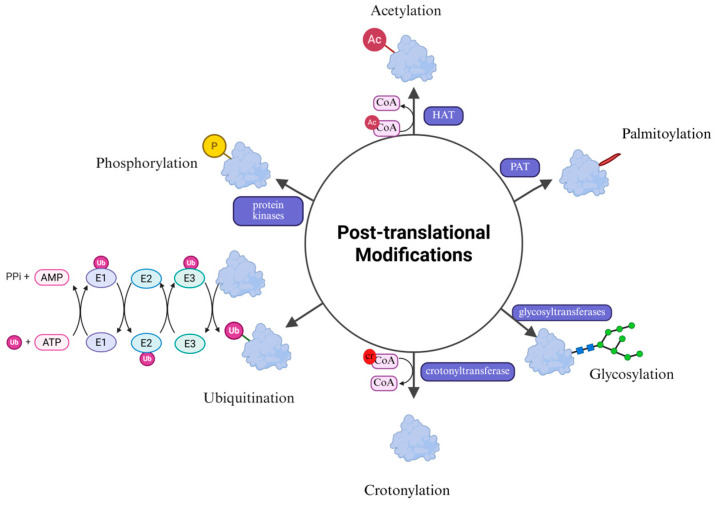
Common post-translational modifications. The main types of post-translational modifications of proteins include acetylation, glycosylation, phosphorylation, ubiquitination, palmitoylation and crotonylation. These modifications finely regulate the biological functions of proteins by altering their structure, stability, localization and interactions.

PTMs are crucial for the regulation of protein activity, localization, expression, and interaction with other cellular molecules [23]. This process involves the addition or removal of specific chemical groups to amino acid residues of proteins to alter their function and localization, thus enriching the proteome [24]. This plays a key role in multiple physiological and cellular processes, including cell differentiation [25], protein degradation, signal transduction and regulation, regulation of gene expression, and protein-protein interactions [26]. To date, over 200 PTMs have been identified [27]. These chemical modifications, such as phosphorylation, glycosylation, and ubiquitination, dynamically regulate protein homeostasis and modulate diverse cellular signals in response to environmental changes [28], with wide-ranging implications for research (Figure 2).

PTMs are crucial for ensuring the normal biological functions of proteins and are consequently involved in almost all biological processes. It is therefore perhaps unsurprising that a rich array of PTMs is also involved in the various viral and cellular processes of HIV infection. These chemical modifications are hijacked by the virus when overtaking the host and also contribute to the cellular response to viral infection [29]. Furthermore, the virus effectively remodels host cellular pathways through PTMs to replicate and evade immune defenses [30]. At present, research on HIV-related PTMs is showing a continuous growth trend [20]. However, there is still a lack of systematic reviews on this topic. Here, we summarize recent research on various HIV-related PTMs, such as phosphorylation, acetylation, glycosylation, ubiquitination, palmitoylation and crotonylation. To gain a deeper understanding of the potential of different PTMs for early diagnosis and treatment, this review discusses the mechanisms of action of different PTMs in HIV infection.

## 2. PTMs and HIV

### 2.1. Acetylation

**Figure 3:**
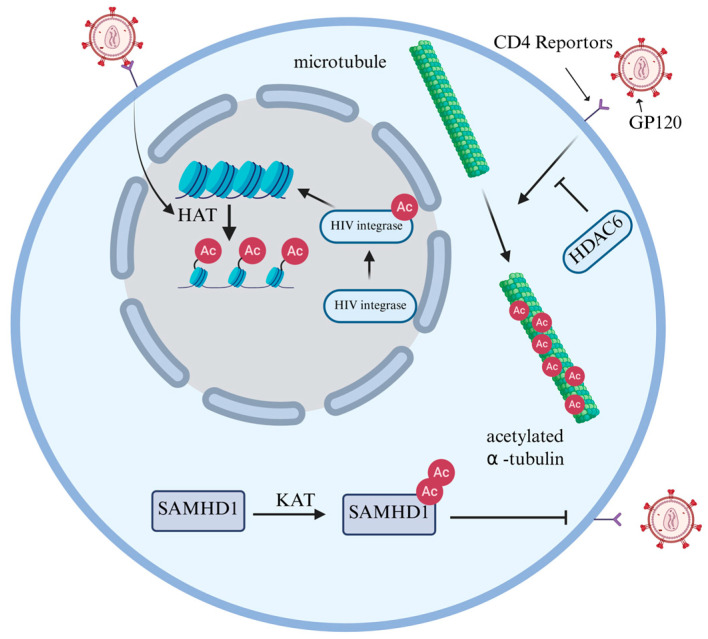
The regulatory role of histone and non-histone acetylation in HIV infection. The binding of GP120 to CD4+ induces acetylated α-tubulin formation, which stabilizes microtubules and facilitates HIV infection. However, HDAC6 inhibits tubulin acetylation and suppresses infection. Additionally, acetylation enhances IN by increasing its DNA affinity and improving strand transfer activity. HIV infection enhances HAT activity, promoting histone acetylation and weakening the force binding histones to DNA, thereby facilitating transcription of both host and viral genes. SAMHD1 acetylation may alter protein conformation, enabling resistance to HIV-1 infection.

Lysine acetylation is a ubiquitous PTM that is widely present throughout the proteome and helps regulate various cellular functions to maintain homeostasis [31]. It is primarily regulated by two major classes of enzymes: Histone acetyltransferases (HATs) and Histone deacetylases (HDACs) [32]. HATs, including the GCN5 family, p300 family, MYST family and unclassified HATs, catalyze the transfer of acetyl groups to lysine residues [33]. In contrast to HATs, HDACs catalyze the removal of acetyl groups and are categorized into two major classes: Zn^2+^-dependent classical histone deacetylases and NAD-dependent Sirtuin deacetylases (SIRTs) [34]. Histone acetylation requires the transfer of an acetyl group from acetyl-coenzyme A to the ε-amino group of the lysine side chain within the substrate [35]. This reduces the positive charge on histones, diminishing their affinity for negatively charged DNA, thus leading to a relaxation of the nucleosome structure. Consequently, this facilitates the specific binding of various transcription factors to DNA binding sites and activates gene transcription [36]. Regulating histone acetylation constitutes a crucial pathway for HIV to achieve its own transcription and replication goals within the host. The binding of GP120 protein to CD4^+^ induces the formation of acetylated α-tubulin, which contributes to the stability of microtubules, thereby promoting HIV infection [37]. Conversely, it was found that HDAC6 can counteract the acetylation of tubulin, thereby inhibiting HIV infection [38]. Furthermore, acetylation can also modify integrase (IN) to increase its affinity for DNA and enhance its ability to transfer DNA strands [20] (Figure 3).

HIV reshapes chromatin by influencing the expression levels of HAT and HDAC in the host, thereby controlling its own gene transcription. Marimani et al. [39] comparatively analyzed the expression of acetylation-related genes and proteins in HIV-1-positive untreated patients, multi-drug resistant (MDR) TB-HIV co-infected treated patients, and healthy controls. It was found that at the mRNA levels, HAT and HDAC were highest in untreated HIV-1-infected individuals. However, in MDR-TB-HIV co-infected patients who received anti-tuberculosis and ART treatments, their expression levels were significantly inhibited, and were reduced to levels between those of healthy individuals and the HIV single infection group. These changes in protein expression were consistent with the corresponding mRNA levels. The protein levels of HAT1 and HDAC5 significantly increased in the untreated HIV group and decreased after treatment. Functionally, the upregulation of acetylation is related to the fact that viral replication is not inhibited. It therefore stands to reason that HIV loosens the chromatin structure by enhancing HAT activity, promoting the transcription of relevant genes. At the same time, the upregulation of HDAC may be the result of negative feedback regulation (Figure 3). These experiments revealed for the first time that HIV-1 reshapes the host epigenome through HAT/HDAC dysregulation, providing a molecular basis for the development of ‘epigenetic immunotherapy’.

In addition to histones, the acetylation of other proteins is equally crucial for HIV infection. Although acetylation was initially identified as a modification of histones within the cell nucleus, it also occurs on several non-histones, playing a vital role in the regulation of cellular responses and signal transduction [40]. Sterile alpha motif (SAM) domain- and histidine-aspartic acid (HD) domain-containing protein 1 (SAMHD1) is a dNTPase that can reduce dNTP levels in non-circulating cells [41]. This effectively limits HIV-1 infection in non-dividing cells (such as macrophages, dendritic cells and resting CD4^+^ T cells) [42]. Bulnes-Ramos et al. [43] conducted proteomic scans on endogenous SAMHD1 from cycling THP-1 cells, non-cycling THP-1 cells, primary B cells, and macrophages, which revealed that it could be acetylated at three lysine sites: K354, K494, and K580. Among them, K580 acetylation was significantly enriched in non-dividing cells (macrophages). Subsequently, when the researchers mutated K580 to arginine (R), glutamine (Q), alanine (A), or glutamic acid (E), SAMHD1 completely lost its ability to block HIV-1 infection, while K354 and K494 mutants did not lose this ability. The absence of K580 acetylation does not affect the nuclear localization of SAMHD1, the activity of dNTP enzymes, or the tetramerization induced by dNTPs. However, detection with specific antibodies revealed that the proportion of K580 acetylation was significantly higher in macrophages than in monocytes, and gradually increased during their differentiation process. These results indicated that K580 acetylation is a key molecular switch in the effect of SAMHD1 against HIV-1 infection. Its action is independent of the classical dNTP-enzyme pathway and may achieve viral restriction by regulating protein conformation, providing a new target for anti-HIV strategies targeting the acetylation pathway (Figure 3).

### 2.2. Glycosylation

Protein glycosylation refers to the covalent attachment of sugar groups (such as monosaccharides, oligosaccharides or polysaccharides) to specific amino acid residues, which represents one of the most abundant PTMs [44]. Accordingly, it plays a key role in protein folding, stability and function [45]. The glycosylation process involves numerous specific glycosyltransferases (GTs), glycosidases, sugar-nucleotide transporters and protein chaperones, so that the final glycan structure depends on an interplay between a number of diverse molecular actors [46]. The glycosylation of proteins is catalyzed by GTs, which transfer sugar moieties from an activated donor molecule such as activated nucleotide sugars or phospholipid-linked sugars [47]. Glycosylation plays a significant role in signal recognition, and HIV achieves immune escape through abundant glycosylation of the virion surface. Furthermore, HIV relies on the host’s glycosylation machinery for correct protein folding, and specific N-linker glycans are crucial for the correct integration of gp160 into viral particles. The absence of these glycans seems to lead to the misfolding of trimer envelope proteins, preventing them from being effectively secreted and displayed on the viral surface, highlighting the crucial role of N-linked glycans [48].

**Figure 4:**
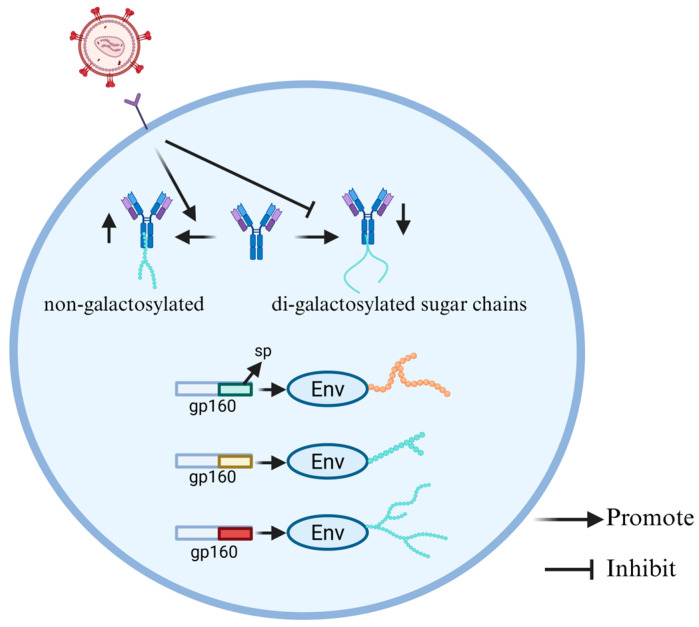
HIV-associated alterations in glycosylation patterns of Env and IgG. The different sps in the precursor protein gp160 of the envelope protein can cause different glycosylation of Env. HIV infection leads to a greater number of non-galactosylated and fewer di-galactosylated sugar chains in the IgG glycosylation pattern.

The HIV-1 envelope protein contains approximately 90 N-glycosylation sites, which play a significant role in determining viral sensitivity to neutralizing antibodies (nAbs) [49], which directly block the ability of pathogens to infect host cells [50]. The HIV-1 envelope protein is the only site on the viral surface that can be recognized by nAbs. Its precursor protein gp160 contains a signal peptide (SP) for targeting envelope (Env) to the endoplasmic reticulum (ER) [51]. Although the SP is not retained in the mature protein, its sequence diversity has a significant impact on the glycosylation and function of Env. Upadhyay et al. [52] constructed a series of SP-exchanged viruses and analyzed the changes in the glycosylation patterns of the Env protein. The results showed that significant changes occurred in the binding of the SP-exchanged Env proteins with mannose- and fucose-binding lectins, indicating a change in the mode of glycosylation (Figure 4). Further analysis revealed that SP exchange at multiple N-glycosylation sites led to changes in sugar chain types (such as complex and oligosaccharide types), particularly in the V1V2 region at the top of the Env trimer and the N88 site at the trimer base. This also reveals how glycosylation of Env contributes to the immune escape of HIV-1 [52].

There are significant differences in the IgG Fc glycosylation patterns during HIV infection between progressor children and pediatric non-progressors (PNPs), and these differences are closely related to the level of immune activation and disease control. IgG is one of the most abundant immunomodulatory proteins in human serum, primarily produced in response to infection, vaccination, or autoimmunity [53]. The glycosylation of the IgG Fc domain is the core mechanism for the functional diversification of antibodies. By altering the balance of type I and II Fcγ receptors (FcγR), the core Fc glycan can be changed, thereby influencing the function of antibodies [54]. Core Fc glycans can be modified by different sugars, including core fucose (Fuc), N-acetylglucosamine (GlcNAc), galactose (Gal), and in the presence of galactose, N-acetylneuraminic acid (NeuAc) or sialic acid [55]. Muenchhoff et al. [56] conducted a systematic immunological analysis on progressor children and PNPs infected with HIV. They studied the Fc glycosylation pattern of IgG antibodies and purified those specific for HIV p24 and gp120 to analyze their glycosylation characteristics. Notably, the IgG glycosylation pattern of progressors shows more non-galactosylated and fewer di-galactosylated sugar chains, while the IgG glycosylation pattern of PNPs was similar to that of HIV-negative children, indicating a lower level of immune activation. The fundamental reason for this difference lies in the disparity of immune activation levels. Progressors remain in a state of high immune activation and chronic inflammation for a long time, inducing the expression remodeling of B cell glycosyltransferase, thereby forming an inflammatory-type IgG glycosylation profile characterized by increased non-galactosylated and decreased di-galactosylation (Figure 4). In contrast, PNPs have a consistently lower immune activation level, maintaining an IgG glycosylation pattern similar to that of uninfected children, with stable immune characteristics. In addition, in HIV-infected children, the fucosylation level of GP120-specific IgG was higher than that of P24-specific IgG, and the sialylation level of GP120-specific IgG was higher than that of total IgG. The unique glycosylation pattern of gp120-specific IgG stems from the antigen-specific immune regulation. The strong Tfh-IL-21 response and highly active germinal center reaction induced by the gp120 antigen have restructured the Fc glycosylation program of B cells, resulting in a higher level of sialylation of gp120-specific IgG; this process promotes the deposition of immune complexes in the germinal center and the affinity maturation of antibodies, thus the sialylation level of gp120-specific IgG is significantly higher than that of total IgG. The IgG glycosylation pattern of progressive patients shows more inflammation-related features, while the IgG glycosylation pattern of PNPs was largely comparable to that of healthy controls, which is consistent with the lower immune activation level of PNPs. These studies provide a new perspective on the development of HIV vaccines for children [56].

### 2.3. Phosphorylation

Protein phosphorylation is a prominent reversible PTM involving the addition of phosphate groups to amino acid side chains. This process is mediated by protein kinases, whilst the reverse process of dephosphorylation is catalyzed by phosphatases [57]. Protein phosphorylation is a key mechanism that regulates the functions of proteins as well as the cell as a whole, and is involved in almost all life processes, including cell division, signal transduction and regulation of gene expression, which are closely related to pathogen infection [58]. Phosphorylation most commonly occurs on serines, followed by threonines and tyrosines [59]. During the phosphorylation process, kinases catalyze the transfer of γ-phosphate groups to the nucleophilic center of the receptor molecule or molecules such as phosphoinositol [60]. Adding negatively charged phosphate groups to proteins will affect their conformation, which has a profound impact on protein activity, stability, interactions and localization [61]. In the life cycle of HIV-1, the phosphorylation status of host and viral proteins plays a decisive role. The HIV Env gp120 induces phosphorylation of the lymphocyte-specific protein tyrosine kinase (LCK), a member of the steroid receptor coactivator (SRC) family [62]. In addition, it also stimulates the nuclear translocation of transcription factor p65 (RELA, also known as NF-κB) [63], which is necessary for the later phase of long terminal repeat (LTR)-dependent transcription of the viral genome. LCK activation induces the phosphorylation of tyrosine kinase ZAP70 and many cellular substrates, leading to the depletion of F-actin in the subsynaptic cortex, which promotes the transmission of the virus from the core to the nucleus after the virus enters [64]. In addition, phosphorylation has been proven to regulate the activity of viral reverse transcriptase (RT). For instance, cyclin-dependent kinase 2 (CDK2) directly phosphorylates RT on the threonine residue T216, thereby enhancing its efficacy and stability [65]. In 2017, Takeuchi et al. determined that maternal embryonic leucine zipper kinase (MELK) is responsible for the phosphorylation of serine S149, a modification that seems crucial for the synthesis of viral cDNA [66].

**Figure 5:**
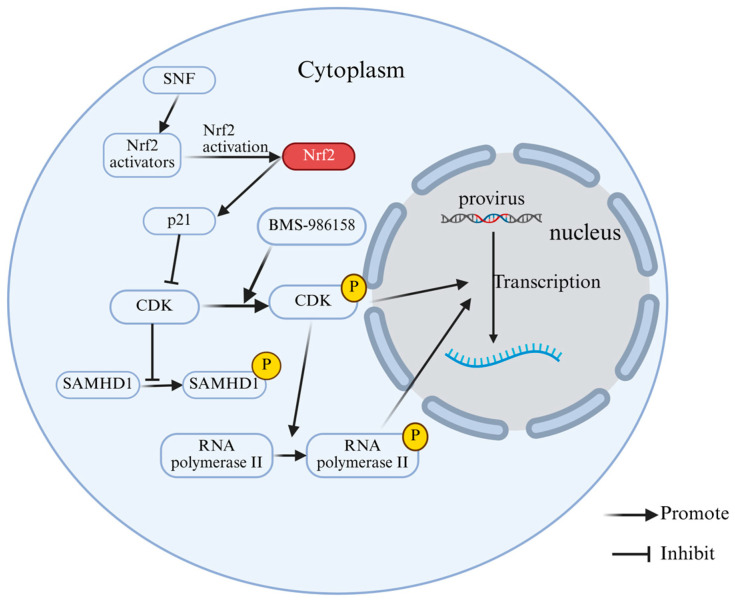
Phosphorylation modification regulates the function of HIV latent reversal and limiting factors. BMS-986158 promotes the phosphorylation of CDK proteins. The phosphorylated CDK9 subunit phosphorylates the CTD of RNAP II. Phosphorylated, activated CDKs and phosphorylated RNAP II are efficiently recruited to the LTR promoter region of latent HIV-1 proviruses, thereby promoting transcription. SNF activates Nrf2, leading to p21 upregulation. As a CDK inhibitor, p21 suppresses CDK activity, thereby reducing SAMHD1 phosphorylation levels.

Phosphorylation of proteins can drive the latency reversal of HIV. As a class of conserved transcriptional regulators distinguished by tandem bromodomains, conserved domains and outer terminal domains, the bromodomain and extra-terminal domain (BET) protein family has received increasing attention [67,68]. The BET protein BRD4, which has been thoroughly studied, is regarded as the main obstacle to latency-reversal due to its inhibitory effect on the trans activation of Tat [69]. Positive transcription elongation factor b (P-TEFb) is composed of two subunits, cyclin T1 and CDK9, which play an important role in the transcription of HIV [70]. After Tat is recruited to the HIV-1 LTR, the phosphorylated CDK9 subunit of P-TEFb promotes the transcriptional extension of HIV-1 by phosphorylating the carboxy-terminal domain (CTD) of RNA polymerase II (RNAP II) [71]. MS-986158, a BET inhibitor, activates latent HIV-1 infection by regulating P-TEFb [72]. Huang et al. [73] found that BMS-986158 induced significant phosphorylation of threonine at position 186 of the CDK9 protein in a latent cell model of HIV-1 infection, and the effect was dose-dependent. It also activated the P-TEFb complex. Further experiments confirmed that BMS-986158 treatment promoted the efficient recruitment of phosphorylated, and thus activated, CDK9 and RNAP II to the LTR promoter region of latent HIV-1 proviruses. This confirmed that BMS-986158 promotes the transcription elongation by relieving BRD4 inhibition of P-TEFb, thereby enhancing CDK9 phosphorylation and recruiting P-TEFb/RNAP II to the HIV-1 LTR (Figure 5). These findings establish the phosphorylation of CDK9 at Thr186 as the core molecular mechanism and key regulatory point through which BMS-986158 exerts its potent latency-reversing effect. This positions BMS-986158 as a potential therapeutic candidate for flushing out latent reservoirs of HIV-1 for a true clinical cure [73].

The anti-HIV-1 activity of SAMHD1 is strictly regulated by the phosphorylation status at its T592 site [74,75]. Dephosphorylated SAMHD1 exhibits stronger viral restriction activity. The Nuclear factor erythroid 2-related factor 2 (Nrf2) pathway is an important upstream signaling axis that regulates the phosphorylation status of SAMHD1 [76]. Studies have found that Sulforaphane (SFN), as a classic Nrf2-mobilizing, significantly enhances Nrf2 activity in human monocyte-derived macrophages (hMDMs) and reduces the phosphorylation level of SAMHD1 at the T592 site in a dose-dependent manner, without affecting its total protein expression level, thereby significantly inhibiting HIV-1 infection [75]. Mechanistic studies have shown that SFN activates Nrf2 to upregulate the CDK inhibitor p21, inhibits intracellular CDK activity, and thereby reduces the phosphorylation of SAMHD1 at the T592 site, maintaining its antiviral functional state (Figure 5). When SAMHD1 is knocked out or significantly reduced in macrophages, SFN loses its inhibitory effect on HIV-1, further demonstrating that its antiviral effect strictly depends on the presence of SAMHD1. This was the first study to directly associate the Nrf2 pathway with the phosphorylation status of SAMHD1, indicating a possible new strategy for regulating its function using Nrf2 agonists to reverse HIV latency and modulate viral restriction [75].

### 2.4. Ubiquitination

The ubiquitin system constitutes a pivotal regulatory hub in HIV-1 infection and host defense. Ubiquitin (Ub) is a small 8.6 kDa protein that can be covalently attached to target proteins as a PTM [77], most commonly via an isopeptide bond between the C-terminal end of Ub and the ε-amino group of a lysine side-chain of the substrate. Histone ubiquitination is significantly different from other histone PTMs because it involves the covalent binding of a protein containing 76 amino acids. As a consequence, ubiquitination requires the sequential action of ubiquitin-activating enzyme (E1), ubiquitin-conjugating enzyme (E2), and ubiquitin ligase (E3) [78], which leads to the formation of different polyUb chains. Conversely, deubiquitinating enzymes (DUBs) are responsible for removing these Ub markers [79]. The ubiquitination process commences with the ATP-dependent activation of the C-terminal glycine residue of Ub by a specific E1. The Ub molecule forms a covalent thioester with the cysteine (Cys) residue of E1, resulting in a linked UBA1~UB intermediate, which then transfers Ub to the catalytic Cys of E2 through a transthiolation reaction. Subsequently, the E3 ubiquitin ligase interacts with the Ub loaded onto E2 and the substrate protein, resulting in the formation of an isopeptide bond between the C-terminus of Ub and a specific Lys residue of the substrate [80]. During the process of HIV-1 infection, the virus actively hijacks the ubiquitination machinery of the host to promote its own replication and immune escape. However, host cells also use ubiquitination to limit viral proliferation. Notably, ubiquitination is involved in the sorting of HIV proteins. For instance, the E3 ubiquitin ligase SH3RF1 associated with the human trans-Golgi network (TGN) has been identified as an important sorting factor for the localization of viral Gag protein to the plasma membrane [81].

**Figure 6:**
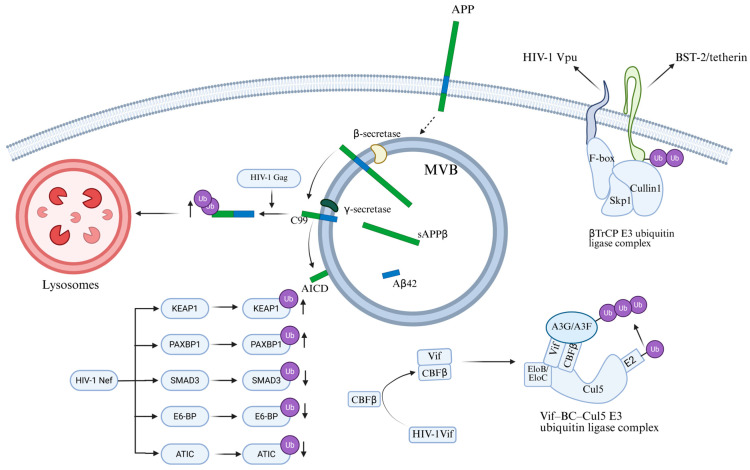
The bidirectional role of the ubiquitination system in HIV replication and host defense. HIV-1 Gag protein promotes polyubiquitination of the C99 C-terminal fragment of APP, leading to increased degradation of C99. HIV-1 Nef upregulates ubiquitination of proteins such as KEAP1 and PAXBP1, while downregulating ubiquitination of proteins including SMAD3, E6-BP, and ATIC. Vif is assembled into the Vif–BC–Cul5 E3 ubiquitin ligase complex, which ubiquitinates and degrades APOBEC3G and APOBEC3F. Vpu recruits β-TrCP to induce the ubiquitination of BST-2/Tetherin, thereby removing BST-2 from the plasma membrane.

HIV plays a crucial role in the pathogenesis, immune evasion and immune regulation by regulating the ubiquitination modification of host factors. The APOBEC3 (A3) family proteins are mammalian-specific cellular deaminases and possess a strong ability to restrain lentivirus replication [82]. When HIV-1 enters a cell, the immune system responds by inducing the activation of the APOBEC3 family proteins [83]. These proteins will convert cytosines to uracils in the intermediate of single-stranded DNA replication, thereby neutralizing the virus [84]. HIV counteracts this intrinsic immune response by encoding a protein known as the viral infectivity factor (Vif). Vif targets A3 to an E3 ubiquitin ligase complex for poly-ubiquitination and proteasomal degradation [85]. Kotaro Shirakawa et al. [86] have conducted research revealing the antagonistic mechanism of HIV-1 Vif protein against various APOBEC3 family antiviral factors. The study found that Vif assembles the Vif–Cullin5–ElonginB–ElonginC (Vif–BC–Cul5) E3 ubiquitin ligase complex to ubiquitinate the APOBEC3 target proteins (Figure 6). During the assembly process, non-canonical cellular cofactor core-binding factor beta (CBFβ) is required to stabilize Vif and its association with the E3 ligase. Only APOBEC3s that can be recognized and bound by Vif (such as APOBEC3G and APOBEC3F) will be ubiquitinated, thereby reducing their levels within the cells and reducing their loading into virus particles; APOBEC3B that cannot bind to Vif is thus tolerant to Vif. This research provides new target ideas for anti-HIV strategies [86]. HIV-1 viral protein U (Vpu) is a protein composed of 81 amino acids, featuring an N-terminal transmembrane domain and a large cytoplasmic domain, which can regulate various proteins [87]. BST-2/tetherin is an interferon-induced restriction factor that can prevent virus release [88]. The Vpu protein binds to BST-2/Tetherin through its transmembrane domain and recruits the E3 ubiquitin ligase complex composed of Skp1-Cullin1-F-box (SCF) protein βTrCP via the S52/56 site in the cytoplasmic tail. This induces poly-ubiquitination of BST-2/Tetherin, thereby blocking its transport to the cell membrane and promoting endocytosis and lysosomal degradation (Figure 6). As a result, the surface expression of BST-2/Tetherin is continuously depleted, enhancing the infectivity and immune evasion of HIV-1 [89]. Andrey A Tokarev et al. [90] have clarified the molecular mechanism by which the HIV-1 Vpu protein antagonizes the host restriction factor BST-2/Tetherin through the β-TrCP-dependent ubiquitination pathway. The experimental results show that Vpu can significantly enhance the ubiquitination modification of BST-2/Tetherin, and this process depends on the interaction between Vpu and β-TrCP. When the β-TrCP binding site of Vpu is mutated, the enhancement effect of Vpu on the ubiquitination of BST-2/Tetherin, the cell surface down-regulation ability, and the relief function of virus release restriction are all significantly impaired. Through systematic mutation analysis of the ubiquitin receptor sites in the cytoplasmic region of BST-2/Tetherin, the study further discovered that although lysine, cysteine, and serine/threonine residues can all participate in the ubiquitination process, the Ser-Thr-Ser (STS) sequence is a key structural element necessary for Vpu’s functional antagonism. Its absence significantly weakens the surface down-regulation ability of Vpu on BST-2/Tetherin and its relief function on HIV release restriction. These findings provide a theoretical basis for developing new antiviral strategies targeting the viral accessory protein-ubiquitin system [90]. HIV-1 viral protein R (Vpr), a small basic 14-kDa protein of 96 amino acids, is a nucleocytoplasmic shuttling protein required for virus replication in vivo [91]. While HIV-1 Vpr plays an essential role in virus replication, the molecular mechanisms underlying its essentiality remain enigmatic. The most clearly defined function of Vpr is the ability to induce the depletion of several dozen host proteins by hijacking a cellular E3-ubiquitin ligase complex [92]. However, Vpr does not enhance the ubiquitination of all proteins within the host cell. Sakshi Arora et al. [93] have conducted research revealing the global regulatory role of HIV-1 Vpr on the host ubiquitination homeostasis. Through whole-cell infection, accessory gene screening, and mutation analysis, the researchers discovered that Vpr is both necessary and sufficient for the decrease in the host’s total cellular ubiquitination level induced by HIV-1 infection. Although Vpr reduces the overall ubiquitination level of most host proteins, it significantly enhances the ubiquitination and degradation of key viral restriction factors such as Uracil-DNA Glycosylase 2 (UNG2) and APOBEC3G. This indicates that Vpr does not inhibit the host ubiquitin-proteasome system but rather reshapes its substrate selectivity, redirecting the limited ubiquitination capacity from ordinary host proteins to virus-advantageous targets, thereby optimizing the clearance efficiency of antiviral restriction factors. This discovery provides a new theoretical framework for understanding how viruses precisely regulate host protein homeostasis to promote their own replication, and expands our understanding of the HIV immune evasion mechanism [93].

HIV-1 hijacks the ubiquitination pathway evade host restriction. Recently, it was discovered that amyloid precursor protein (APP) is highly expressed in macrophages and microglia, where it inhibits HIV-1 replication [94], while HIV-1 promotes APP ubiquitination to achieve effective replication and escape. APP is a transmembrane protein with three main subtypes [95]. APP695 is mainly expressed in neurons, while APP751 and APP770 are the main subtypes expressed in non-neuronal cells. Consisting of a large extracellular domain, a single transmembrane domain and a short cytoplasmic tail, APP is located on the plasma membrane and intracellular vesicles. Gu et al. [94] discovered that the Gag protein of HIV-1 promotes the polyubiquitination of the APP C-terminal fragment C99, thereby regulating the ER-Golgi transport of C99 and its further processing into toxic amyloids. When HEK293A cells were transfected with HIV-1, there was an increase in the degradation of APP and its C-terminal fragments. When cells were treated with the ubiquitination inhibitor TAK-243, the stability of C99 was enhanced, indicating that HIV-1 infection promotes the ubiquitination of C99. Further experiments have shown that mutations affecting the ubiquitination sites of C99 (especially the K724-726A mutation) can significantly restore the stability of C99, and this mutation can prevent Gag from entering CD63-positive multivesicles, thereby inhibiting the release of viral particles (Figure 6). These results indicate that the amyloid processing pathway of APP plays a significant role in sensing and inhibiting HIV-1 replication, while HIV-1 evades this restriction mechanism by promoting the ubiquitination of C99 [94]. This indicates that regulating the ubiquitination of APP may be a potential strategy for inhibiting HIV proliferation.

HIV-1 negative regulatory factor (Nef) plays a key role in determining the fate of cellular proteins by regulating their ubiquitination. Nef is a multifunctional regulatory protein encoded by the viral genome, which plays an important role in the replication, transmission and pathogenicity of HIV [96]. Ghaly et al. [97] found that Nef significantly reduced the overall ubiquitination level of intracellular proteins. The researchers overexpressed the constructs Myc.His-tagged HIV-1 Nef (Nef-Myc) and HA-tagged ubiquitin (Ub-HA) with epitope tags in HEK293T cells, and combined with ubiquitination proteomics analysis. They found that, in the presence of Nef, the ubiquitination status of a total of 325 cellular proteins was significantly altered. Among them, the ubiquitination levels of 93 proteins increased, while those of 232 proteins decreased. Further systematic analysis revealed that these differentially ubiquitinated proteins are primarily involved in binding and catalytic activities, participate in cellular as well as metabolic processes, and are predominantly localized to organelles such as the cytoplasm and cell membrane. This aligns with Nef’s known functions and localisation during HIV-1 infection. Among them, the ubiquitination of Kelch-like ECH-associated protein 1 (KEAP1) increased tenfold in the presence of Nef, while its total protein expression level remained practically unchanged. The ubiquitination of PAXBP1 was increased 36-fold, while its total protein expression level slightly decreased. The ubiquitination of SMAD3 was reduced 3-fold, while its total protein expression level remained unchanged. These results indicate that the effect of Nef on ubiquitination levels is not related to the overall expression levels of proteins, but specifically regulates their ubiquitination status (Figure 6). The research results show that Nef regulates the stability and degradation of intracellular proteins by regulating their ubiquitination state, which is crucial for the replication and pathogenicity of HIV-1 [97].

### 2.5. Palmitoylation

Palmitoylation is a reversible PTM in which fatty acid chains, typically palmitate, are attached to the Cys residues of proteins through reversible thioester bonds [98]. The addition of lipid chains to proteins increases their hydrophobicity and regulates protein stability, interaction with effector proteins, subcellular localization and membrane transport. The S-palmitoylation of proteins is mainly catalyzed by palmitoyl S-acyltransferases (PATs). Mammalian cells contain 23 PAT family members, which have similar structures and all contain a zinc finger DHHC (ZDHHC) domain [99]. ZDHHC catalyzes protein palmitoylation through a two-step process involving its own palmitoylation to form an acyl-enzyme intermediate, followed by transfer of the acyl moiety to the target Cys residue in the substrate protein. In contrast to other forms of protein lipidation, palmitoylation can be reversed by deacylases from the serine hydrolase superfamily [100]. The currently known deacylases include acyl-protein thioesterases 1 and 2 (APT1 and APT2) and protein palmitoyl thioesterase1 (PPT1), as well as AB hydrolase domain-containing 10 (ABHD10), ABHD17A, ABHD17B and ABHD17C [101,102,103]. In recent years, the targeting of palmitoylation to block HIV infection has gradually come into focus. C-C chemokine receptor type 5 (CCR5) is the main co-receptor that is responsible for the transmission and establishment of HIV-1 infection. Studies have shown that inhibiting the palmitoylation of CCR5 can effectively reduce its transport to the plasma membrane, thereby blocking HIV infection [104]. In addition, it was found that the cytoplasmic domain of the HIV-1 Env glycoprotein gp160 contains two palmitoylated Cys residues, and gp160 targets the detergent-insoluble membrane domains through palmitoylation [105].

**Figure 7:**
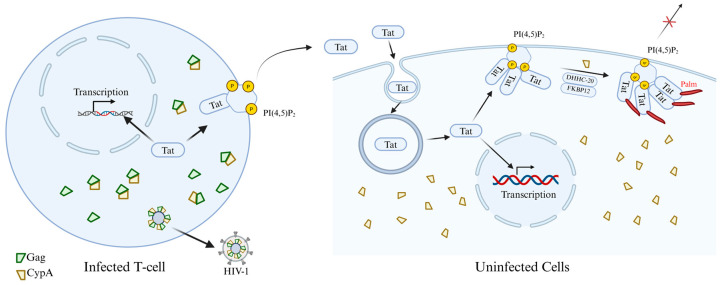
The mechanism by which palmitoylation of Tat protein regulates the pathogenicity of HIV and viral secretion. The newly synthesized Tat protein enters the nucleus to facilitate efficient transcription of viral genes. However, the majority of Tat is secreted from infected cells via an unconventional export mechanism dependent on plasma membrane-associated PI(4,5)P_2_ for its recruitment. Concurrently, Gag multimerization mediates viral assembly at the plasma membrane. Since Gag binds to CypA, viral budding depletes cellular CypA levels, thereby specifically suppressing Tat palmitoylation within infected cells. Secreted Tat enters target cells (such as macrophages, neurons, muscle cells, etc.) via endocytosis. Tat may subsequently enter the nucleus to influence cellular gene transcription; however, the majority recruits to PI(4,5)P_2_ on the plasma membrane, where it undergoes palmitoylation catalyzed by DHHC-20 with the assistance of two proline isomerases, CypA and FKBP12. Tat palmitoylation inhibits its secretion and enables cumulative effects from minute Tat doses, effectively impairing PI(4,5)P_2_-dependent cellular functions.

The HIV-1 Tat protein is precisely regulated by palmitoylation, which determines its subcellular localization and profoundly affects viral replication and transmission, as well as the underlying pathogenic mechanisms. Most HIV-1 Tat is unconventionally secreted by infected cells after interacting with phosphatidylinositol (4,5) bisphosphate (PI(4,5)P_2_) on the plasma membrane. Chopard et al. [106] revealed the mechanism by which the intracellular localization and function of Tat are regulated through protein palmitoylation during infection, thus affecting viral pathogenicity. Research has found that the Tat protein does not undergo palmitoylation in infected cells but is secreted efficiently. However, when secreted Tat reaches neighboring uninfected cells, it can be palmitoylated at the Cys31 site by S-acyltransferase DHHC-20. This modification depends on the binding of Tat to PI(4,5)P_2_ and is assisted by two prolyl isomerases, cyclophilin A (CypA) and FKBP12. Palmitoylation significantly enhances the affinity of Tat for PI(4,5)P_2_, enabling it to stably target the cell membrane, thereby inhibiting its secretion and increasing its accumulation within the cell. Functionally, palmitoylated Tat disrupts PI(4,5)P_2_-dependent membrane transport processes, such as phagocytosis in macrophages and secretory activities in neurons. Further research revealed that the viral Gag protein binds to CypA and incorporates it into viral particles, depleting CypA within infected cells. This inhibits Tat palmitoylation and promotes its secretion (Figure 7). This mechanism ensures that the virus retains Tat during early infection to enhance transcription, while promoting its secretion in the later stages to influence neighboring uninfected cells [106]. This discovery not only deepens our understanding of the pathogenic mechanisms of HIV-1 but also provides a theoretical basis for the development of novel antiviral strategies targeting Tat palmitoylation or its interaction with PI(4,5)P_2_.

### 2.6. Crotonylation

Lysine crotonylation represents a novel PTM of proteins discovered in recent years, exhibiting specific enrichment at active gene promoters and potential enhancers within mammalian nuclei [107]. Significant advances have been made in recent years in research on the regulation of gene expression, cellular metabolism, and disease pathogenesis. Similar to other lysine acylations, crotonylation occurs at the ε-amino group of lysine, neutralizing the positive charge of the residue [108]. The protein crotonylation level in cells is precisely regulated by the activities of reader proteins, crotonyltransferase (writer) and decrotonylase (eraser), but it is also affected by the intracellular concentration of crotonyl-CoA (Cr-CoA) [109]. The crotonyl group is transferred to the lysine residue under the action of histone crotonyltransferase with Cr-CoA as the substrate [110].

**Figure 8:**
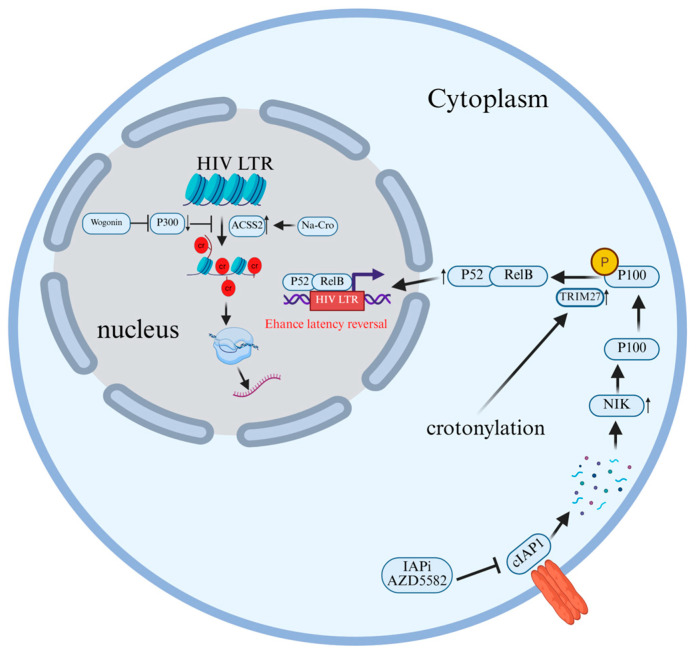
The molecular mechanism by which crotonylation modification promotes the reversal of HIV latency. Sodium crotonate addition induces ACSS2 expression in cells, promoting histone crotonylation in the HIV LTR region and thereby enhancing HIV transcription. When targeted by IAPi, cIAP1 undergoes auto-ubiquitination and proteasomal degradation. The absence of cIAP1 allows NIK to accumulate and phosphorylate p100. Crotonylation enhances TRIM27 activity, promoting cleavage and release of the active p52 subunit, thereby activating the NF-κB pathway and amplifying viral gene transcription. Wogonin inhibits histone crotonylation by suppressing the expression of histone acetyltransferas.

Moreover, recent research revealed that HAT activity is related to crotonylation [111]. At present, most of the enzymes that catalyze histone crotonylation have been identified among HATs [110]. P300/CBP is the dominant histone crotonyltransferase in mammalian cells, where it can promote transcription by increasing histone crotonylation and recruiting AF9/DPF2, which in turn can be reversed by decrotonylase [112]. In addition, HDAC also has histone decrotonylation (HDCR) activity [113]. Since it has been proven that acetylation and crotonylation share writers and erasers, it stands to reason that they might also share readers [114]. In the research on HIV-1 latency and reactivation, crotonylation has been confirmed to be a key driving force for breaking through viral transcriptional blockade and reshaping the chromatin conformation of the provirus. Research indicates that acetyl-CoA synthase short-chain family member 2 (ACSS2) influences HIV replication and latency by regulating histone crotonylation within the HIV LTR region. Elevated crotonylation levels disrupt latency, initiating viral replication, while low crotonylation levels favor viral persistence in the latent state [115] (Figure 8).

Crotonylation reshapes the chromatin conformation in the HIV promoter region, thereby breaking through the latency barrier and activating viral transcription. Zhang et al. [116] found that wogonin could significantly decrease the crotylation level of histones H3/H4 and inhibit the latent reactivation of HIV-1 by specifically inhibiting the expression of HAT p300, without a significant effect on the expression of HDAC1 (Figure 8). Furthermore, the use of the P300-specific small molecule agonist CTPB can reverse the downregulating effect of wogonin on histone crotonylation, thereby restoring the reactivation of HIV-1. This indicates that wogonin inhibits the reactivation of latent HIV-1 provirus by regulating the epigenetic modification mechanism of histone crotonylation, providing a new potential strategy for the functional cure of HIV-1 [116].

Targeting crotonylation has been confirmed as a viable strategy to promote the latency reversal of HIV induced by the inhibitor of apoptosis (IAP)-inhibiting small molecule (IAPi) AZD5582, and its mechanism involves promoting the activation of the non-canonical NF-κB signaling pathway via TRIM27. Li et al. [117] induced crotonylation by adding sodium crotonate (NaCr) to cultured cells and combined it with IAPi (AZD5582) treatment. They found that crotonylation could significantly enhance the HIV latency reversal effect of AZD5582, which manifested as higher viral RNA expression levels and more green fluorescent protein (GFP)-positive cells, which was used as an indicator of HIV transcriptional activity. In addition, the study also found that crotonylation promoted the activation of the ncNF-κB signaling pathway by enhancing the cleavage of p100 to yield p52, which may involve the E3 ubiquitin ligase TRIM27. In addition, the results of siRNA knockdown experiments indicated that the deletion of TRIM27 would reduce p100/p52 levels and inhibit the reversal of HIV latency [117] (Figure 8).

## 3. Summary and Outlook

This paper systematically reviews the regulatory roles of protein PTMs in HIV infection, replication and latency, with a focus on the molecular mechanisms of six key PTM types: acetylation, glycosylation, phosphorylation, ubiquitination, palmitoylation and crotonylation, as well as corresponding intervention strategies targeting different aspects of the viral life cycle. PTMs form a key hub of virus-host interactions, as viruses reshape the epigenetic landscape by hijacking the host’s PTM system (such as acetylation/phosphorylation), thereby driving the latency–reactivation transition. Notably, PTMs are site-specific, so the same modification at different sites of the same protein can have opposite biological effects. Beyond the PTMs discussed herein, several novel PTMs have emerged as additional players during HIV infection. For instance, HIV-1 integrase (IN) undergoes SUMOylation, though its function remains unclear. At the same time, it is not clear which SUMO subtype preferentially targets IN and whether IN is subject to single or multiple SUMOylation [118].

The main obstacle to curing HIV lies in infected long-dormant memory CD4^+^ T cells, which persist during ART [119]. One strategy under study to flush out this latent reservoir is to activate the virus in the presence of HAART. As a consequence, cells may die through immune-mediated clearance or virus-induced cell lysis, thus preventing further infection. Epigenetically modifying agents are small-molecule compounds that target epigenetic processes, such as inhibiting the transfer of methyl, acetyl or alkyl groups, thereby causing epigenetic changes [120]. An increasing number of studies have shown that HDAC inhibitors (HDACi) alter protein acetylation, gene expression patterns and cell fate determination, making them promising new drugs for the treatment of HIV infection. HDACis are classified into the four major structural families of short-chain aliphatic acids, hydroxamic acids, benzamides, and cyclic tetrapeptides and depsipeptides [121]. Furthermore, one functional cure strategy, called “block-and-lock,” has recently gained substantial attention [122]. This therapeutic approach aims to permanently silence all proviruses even after treatment interruption by using latency-promoting agents (LPAs) to steadily suppress the transcription of the viral reservoir and lock the HIV promoter in deep latency [123]. Table 1 summarizes the drugs currently showing potential for application in novel HIV therapies based on these principles.

Although an increasing number of studies have implicated different PTMs in a series of biological processes, existing research mostly focuses on a single class of PTMs, while the crosstalk among them, i.e., the coordination of multiple PTMs, has thus not received sufficient attention. It is therefore necessary to integrate omics technologies to analyze the dynamic interaction map of PTMs and reveal the collaborative control effect of “modified codes” on viral latency. Concurrently, while extensive preclinical research has identified thousands of disease-associated PTMs [135], research into their clinical translation remains underdeveloped. While latency-reversing agents (LRAs) targeting PTMs demonstrate significant potential, their clinical translation faces core challenges such as poor specificity and high toxicity. Gradually accumulating evidence suggests that due to the extensive integration landscape of proviruses, the condensed chromatin state of resting T cells, the sequestration of essential transcription factors, and the physiological heterogeneity of host cells, commonly used LRAs may fail to reactivate the entire HIV-1 reservoir [136]. Future research should therefore focus on developing reservoir-specific precision delivery systems. PTM of proteins is closely related to HIV infection and is a highly active research field. Thus, PTMs offer a new paradigm for a potential radical cure of HIV, which is especially significant as AIDS remains one of the few infectious diseases without a curative therapy [137]. In the future, it will be necessary to promote the translation of “epigenetic-immune” combined therapy from basic research to clinical use through multi-omics integration, precise drug delivery and the application of innovative technologies.

## Figures and Tables

**Table 1:** Therapeutic agents targeting epigenetic modifications for HIV latency reversal and their mechanisms of action.

Drug/Compound Name	Category	Mechanism of Action	Ref
vorinostat	Hydroxamic acids	Inhibits histone deacetylases, increasing chromatin acetylation. Acetylated histones exhibit reduced positive charge, diminishing their affinity for DNA. This unlocks closed HIV promoter regions, thereby activating viral transcription.	[121]
trichostatin A	hydroxamic acids		[124]
Marbostat-100	benzamides		[125]
sodium butyrate	Short chain aliphatic acids		[126]
valproic acid	Short chain aliphatic acids		[126]
panobinostat	Hydroxamic acids		[127]
Butyric acid	Short chain aliphatic acids		[121]
givinostat	Hydroxamic acids		[128]
panobinostat	Hydroxamic acids		[129]
Romidepsin	Cyclic tetrapeptides		[130]
Entinostat	benzamides		[131]
mocetinostat	benzamides		[132]
AZD5582	dimeric peptidomimetic small molecule	AZD5582 induces the auto-ubiquitination of cIAP1 and its degradation via the proteasome, thereby relieving its inhibition on NIK and activating the non-canonical NF-κB signaling pathway. Ultimately enhancing the reactivation of latent HIV. Additionally, this process is enhanced by palmitoylation.	[117]
HR73	Small-molecule heteroaromatic organic compound	Blocking Tat deacetylation prevents it from binding to trans-acting responsive element, CyclinT1 and CDK9 to initiate transcription and thus inhibits latent HIV reactivation	[133]
wogonin	Flavone	Inhibits the reactivation of latent HIV-1 by inhibiting the expression of p300, a histone acetyltransferase, and decreasing the crotonylation of histone H3/H4 in the HIV-1 promoter region.	[116]
Senexin A	Small-molecule synthetic heterocyclic compound	By inhibiting the phosphorylation of the transcriptional activator mediated by CDK8/19, the assembly of RNA polymerase II to the HIV promoter region is prevented, thereby inhibiting the reactivation of latent HIV.	[134]
BRD6989	Small-molecule synthetic heteroaromatic compound		[134]

## Data Availability

No new data were created or analyzed in this study.

## References

[1] Masenga S.K., Mweene B.C., Luwaya E., Muchaili L., Chona M., Kirabo A. (2023). HIV-Host Cell Interactions. Cells.

[2] Chou T.C., Maggirwar N.S., Marsden M.D. (2024). HIV Persistence, Latency, and Cure Approaches: Where Are We Now?. Viruses.

[3] Yin X., Langer S., Zhang Z., Herbert K.M., Yoh S., König R., Chanda S.K. (2020). Sensor Sensibility-HIV-1 and the Innate Immune Response. Cells.

[4] Chen J., Zhou T., Zhang Y., Luo S., Chen H., Chen D., Li C., Li W. (2022). The reservoir of latent HIV. Front. Cell. Infect. Microbiol..

[5] Cohn L.B., Chomont N., Deeks S.G. (2020). The Biology of the HIV-1 Latent Reservoir and Implications for Cure Strategies. Cell Host Microbe.

[6] German Advisory Committee Blood (Arbeitskreis Blut), Subgroup ‘Assessment of Pathogens Transmissible by Blood’ (2016). Human Immunodeficiency Virus (HIV). Transfus. Med. Hemotherapy.

[7] Nuwagaba J., Li J.A., Ngo B., Sutton R.E. (2025). 30 years of HIV therapy: Current and future antiviral drug targets. Virology.

[8] Saunders K.O., Counts J., Thakur B., Stalls V., Edwards R., Manne K., Lu X., Mansouri K., Chen Y., Parks R. (2024). Vaccine induction of CD4-mimicking HIV-1 broadly neutralizing antibody precursors in macaques. Cell.

[9] Xiao T., Cai Y., Chen B. (2021). HIV-1 Entry and Membrane Fusion Inhibitors. Viruses.

[10] Negi G., Sharma A., Dey M., Dhanawat G., Parveen N. (2022). Membrane attachment and fusion of HIV-1, influenza A, and SARS-CoV-2: Resolving the mechanisms with biophysical methods. Biophys. Rev..

[11] Sapp N., Burge N., Cox K., Prakash P., Balasubramaniam M., Thapa S., Christensen D., Li M., Linderberger J., Kvaratskhelia M. (2022). HIV-1 Preintegration Complex Preferentially Integrates the Viral DNA into Nucleosomes Containing Trimethylated Histone 3-Lysine 36 Modification and Flanking Linker DNA. J. Virol..

[12] Blanco-Rodriguez G., Gazi A., Monel B., Frabetti S., Scoca V., Mueller F., Schwartz O., Krijnse-Locker J., Charneau P., Di Nunzio F. (2020). Remodeling of the Core Leads HIV-1 Preintegration Complex into the Nucleus of Human Lymphocytes. J. Virol..

[13] Rozina A., Anisenko A., Kikhai T., Silkina M., Gottikh M. (2022). Complex Relationships between HIV-1 Integrase and Its Cellular Partners. Int. J. Mol. Sci..

[14] Elliott J.L., Kutluay S.B. (2020). Going beyond Integration: The Emerging Role of HIV-1 Integrase in Virion Morphogenesis. Viruses.

[15] Freed E.O. (2015). HIV-1 assembly, release and maturation. Nat. Rev. Microbiol..

[16] Ono A., Freed E.O. (2004). Cell-type-dependent targeting of human immunodeficiency virus type 1 assembly to the plasma membrane and the multivesicular body. J. Virol..

[17] Gamble T.R., Yoo S., Vajdos F.F., von Schwedler U.K., Worthylake D.K., Wang H., McCutcheon J.P., Sundquist W.I., Hill C.P. (1997). Structure of the carboxyl-terminal dimerization domain of the HIV-1 capsid protein. Science.

[18] Kleinpeter A.B., Freed E.O. (2020). HIV-1 Maturation: Lessons Learned from Inhibitors. Viruses.

[19] Adamson C.S., Freed E.O. (2010). Novel approaches to inhibiting HIV-1 replication. Antivir. Res..

[20] Chen L., Keppler O.T., Schölz C. (2018). Post-translational Modification-Based Regulation of HIV Replication. Front. Microbiol..

[21] Ling L., Kim M., Soper A., Kovarova M., Spagnuolo R.A., Begum N., Kirchherr J., Archin N., Battaglia D., Cleveland D. (2024). Analysis of the effect of HDAC inhibitors on the formation of the HIV reservoir. mBio.

[22] Tibebe H., Marquez D., McGraw A., Gagliardi S., Sullivan C., Hillmer G., Narayan K., Izumi C., Keating A., Izumi T. (2025). Targeting Latent HIV Reservoirs: Effectiveness of Combination Therapy with HDAC and PARP Inhibitors. Viruses.

[23] Li Y., Zhang R., Hei H. (2023). Advances in post-translational modifications of proteins and cancer immunotherapy. Front. Immunol..

[24] Wang R., Wang G., Qin Z.H. (2019). Protein Modification and Autophagy Activation. Autophagy: Biology and Diseases.

[25] Grotenbreg G., Ploegh H. (2007). Chemical biology: Dressed-up proteins. Nature.

[26] Yang S., Sun Y., Yu W. (2025). HDACs and Their Inhibitors on Post-Translational Modifications: The Regulation of Cardiovascular Disease. Cells.

[27] Keenan E.K., Zachman D.K., Hirschey M.D. (2021). Discovering the landscape of protein modifications. Mol. Cell.

[28] Li Z., Chen J., Huang H., Zhan Q., Wang F., Chen Z., Lu X., Sun G. (2024). Post-translational modifications in diabetic cardiomyopathy. J. Cell. Mol. Med..

[29] Bussienne C., Marquet R., Paillart J.C., Bernacchi S. (2021). Post-Translational Modifications of Retroviral HIV-1 Gag Precursors: An Overview of Their Biological Role. Int. J. Mol. Sci..

[30] Johnson J.R., Crosby D.C., Hultquist J.F., Kurland A.P., Adhikary P., Li D., Marlett J., Swann J., Hüttenhain R., Verschueren E. (2022). Global post-translational modification profiling of HIV-1-infected cells reveals mechanisms of host cellular pathway remodeling. Cell Rep..

[31] Pan S., Chen R. (2022). Pathological implication of protein post-translational modifications in cancer. Mol. Asp. Med..

[32] Song H., Zhang M., Guo C., Guo X., Ma Y., Ma Y. (2025). Implication of protein post translational modifications in gastric cancer. Front. Cell Dev. Biol..

[33] Xue M., Feng T., Chen Z., Yan Y., Chen Z., Dai J. (2022). Protein Acetylation Going Viral: Implications in Antiviral Immunity and Viral Infection. Int. J. Mol. Sci..

[34] Liu W., Yuan Q., Cao S., Wang G., Liu X., Xia Y., Bian Y., Xu F., Chen Y. (2023). Review: Acetylation mechanisms and targeted therapies in cardiac fibrosis. Pharmacol. Res..

[35] Ashton A.W., Dhanjal H.K., Rossner B., Mahmood H., Patel V.I., Nadim M., Lota M., Shahid F., Li Z., Joyce D. (2024). Acetylation of nuclear receptors in health and disease: An update. FEBS J..

[36] Shvedunova M., Akhtar A. (2022). Modulation of cellular processes by histone and non-histone protein acetylation. Nat. Rev. Mol. Cell Biol..

[37] Sabo Y., Walsh D., Barry D.S., Tinaztepe S., de Los Santos K., Goff S.P., Gundersen G.G., Naghavi M.H. (2013). HIV-1 induces the formation of stable microtubules to enhance early infection. Cell Host Microbe.

[38] Valenzuela-Fernández A., Alvarez S., Gordon-Alonso M., Barrero M., Ursa A., Cabrero J.R., Fernández G., Naranjo-Suárez S., Yáñez-Mo M., Serrador J.M. (2005). Histone deacetylase 6 regulates human immunodeficiency virus type 1 infection. Mol. Biol. Cell.

[39] Marimani M., Ahmad A., Stacey S., Duse A. (2020). Examining the levels of acetylation, DNA methylation and phosphorylation in HIV-1 positive and multidrug-resistant TB-HIV patients. J. Glob. Antimicrob. Resist..

[40] Kabir F., Atkinson R., Cook A.L., Phipps A.J., King A.E. (2022). The role of altered protein acetylation in neurodegenerative disease. Front. Aging Neurosci..

[41] Goldstone D.C., Ennis-Adeniran V., Hedden J.J., Groom H.C., Rice G.I., Christodoulou E., Walker P.A., Kelly G., Haire L.F., Yap M.W. (2011). HIV-1 restriction factor SAMHD1 is a deoxynucleoside triphosphate triphosphohydrolase. Nature.

[42] Bowen N.E., Oo A., Kim B. (2022). Mechanistic Interplay between HIV-1 Reverse Transcriptase Enzyme Kinetics and Host SAMHD1 Protein: Viral Myeloid-Cell Tropism and Genomic Mutagenesis. Viruses.

[43] Bulnes-Ramos A., Schott K., Rabinowitz J., Luchsinger C., Bertelli C., Miyagi E., Yu C.H., Persaud M., Shepard C., König R. (2024). Acetylation of SAMHD1 at lysine 580 is crucial for blocking HIV-1 infection. mBio.

[44] Chatham J.C., Patel R.P. (2024). Protein glycosylation in cardiovascular health and disease. Nat. Rev. Cardiol..

[45] Hao C., Zou Q., Bai X., Shi W. (2025). Effect of glycosylation on protein folding: From biological roles to chemical protein synthesis. iScience.

[46] Groux-Degroote S., Cavdarli S., Uchimura K., Allain F., Delannoy P., Donev R. (2020). Glycosylation changes in inflammatory diseases. Advances in Protein Chemistry and Structural Biology.

[47] Liu Z., Yang J., Du M., Xin W. (2023). Functioning and mechanisms of PTMs in renal diseases. Front. Pharmacol..

[48] Watanabe Y., Bowden T.A., Wilson I.A., Crispin M. (2019). Exploitation of glycosylation in enveloped virus pathobiology. Biochim. Biophys. Acta (BBA)-Gen. Subj..

[49] Newby M.L., Allen J.D., Crispin M. (2024). Influence of glycosylation on the immunogenicity and antigenicity of viral immunogens. Biotechnol. Adv..

[50] Wagh K., Seaman M.S. (2023). Divide and conquer: Broadly neutralizing antibody combinations for improved HIV-1 viral coverage. Curr. Opin. HIV AIDS.

[51] Lambert G.S., Upadhyay C. (2021). HIV-1 Envelope Glycosylation and the Signal Peptide. Vaccines.

[52] Upadhyay C., Feyznezhad R., Cao L., Chan K.W., Liu K., Yang W., Zhang H., Yolitz J., Arthos J., Nadas A. (2020). Signal peptide of HIV-1 envelope modulates glycosylation impacting exposure of V1V2 and other epitopes. PLoS Pathog..

[53] Archer E.J., Gonzalez J.C., Ghosh D., Mellins E.D., Wang T.T. (2022). Harnessing IgG Fc glycosylation for clinical benefit. Curr. Opin. Immunol..

[54] Wang T.T., Ravetch J., Nimmerjahn F. (2019). IgG Fc Glycosylation in Human Immunity. Fc Mediated Activity of Antibodies.

[55] Yang S., Cui M., Liu Q., Liao Q. (2022). Glycosylation of immunoglobin G in tumors: Function, regulation and clinical implications. Cancer Lett..

[56] Muenchhoff M., Chung A.W., Roider J., Dugast A.S., Richardson S., Kløverpris H., Leslie A., Ndung’u T., Moore P., Alter G. (2020). Distinct Immunoglobulin Fc Glycosylation Patterns Are Associated with Disease Nonprogression and Broadly Neutralizing Antibody Responses in Children with HIV Infection. mSphere.

[57] Newcombe E.A., Delaforge E., Hartmann-Petersen R., Skriver K., Kragelund B.B. (2022). How phosphorylation impacts intrinsically disordered proteins and their function. Essays Biochem..

[58] Liu X., Zhang Y., Wang Y., Yang M., Hong F., Yang S. (2021). Protein Phosphorylation in Cancer: Role of Nitric Oxide Signaling Pathway. Biomolecules.

[59] Bilbrough T., Piemontese E., Seitz O. (2022). Dissecting the role of protein phosphorylation: A chemical biology toolbox. Chem. Soc. Rev..

[60] Tigno-Aranjuez J.T., Abbott D.W. (2012). Ubiquitination and phosphorylation in the regulation of NOD2 signaling and NOD2-mediated disease. Biochim. Biophys. Acta.

[61] Hong X., Lv J., Li Z., Xiong Y., Zhang J., Chen H.F. (2023). Sequence-based machine learning method for predicting the effects of phosphorylation on protein-protein interactions. Int. J. Biol. Macromol..

[62] Deng J., Mitsuki Y.Y., Shen G., Ray J.C., Cicala C., Arthos J., Dustin M.L., Hioe C.E. (2016). HIV Envelope gp120 Alters T Cell Receptor Mobilization in the Immunological Synapse of Uninfected CD4 T Cells and Augments T Cell Activation. J. Virol..

[63] Flory E., Weber C.K., Chen P., Hoffmeyer A., Jassoy C., Rapp U.R. (1998). Plasma membrane-targeted Raf kinase activates NF-kappaB and human immunodeficiency virus type 1 replication in T lymphocytes. J. Virol..

[64] Len A.C.L., Starling S., Shivkumar M., Jolly C. (2017). HIV-1 Activates T Cell Signaling Independently of Antigen to Drive Viral Spread. Cell Rep..

[65] Leng J., Ho H.P., Buzon M.J., Pereyra F., Walker B.D., Yu X.G., Chang E.J., Lichterfeld M. (2014). A cell-intrinsic inhibitor of HIV-1 reverse transcription in CD4(+) T cells from elite controllers. Cell Host Microbe.

[66] Takeuchi H., Saito H., Noda T., Miyamoto T., Yoshinaga T., Terahara K., Ishii H., Tsunetsugu-Yokota Y., Yamaoka S. (2017). Phosphorylation of the HIV-1 capsid by MELK triggers uncoating to promote viral cDNA synthesis. PLoS Pathog..

[67] Alleboina S., Aljouda N., Miller M., Freeman K.W. (2021). Therapeutically targeting oncogenic CRCs facilitates induced differentiation of NB by RA and the BET bromodomain inhibitor. Mol. Ther. Oncolytics.

[68] Kong B., Zhu Z., Li H., Hong Q., Wang C., Ma Y., Zheng W., Jiang F., Zhang Z., Ran T. (2022). Discovery of 1-(5-(1H-benzo[d]imidazole-2-yl)-2,4-dimethyl-1H-pyrrol-3-yl)ethan-1-one derivatives as novel and potent bromodomain and extra-terminal (BET) inhibitors with anticancer efficacy. Eur. J. Med. Chem..

[69] Conrad R.J., Fozouni P., Thomas S., Sy H., Zhang Q., Zhou M.M., Ott M. (2017). The Short Isoform of BRD4 Promotes HIV-1 Latency by Engaging Repressive SWI/SNF Chromatin-Remodeling Complexes. Mol. Cell.

[70] Egloff S. (2021). CDK9 keeps RNA polymerase II on track. Cell. Mol. Life Sci..

[71] Asamitsu K., Fujinaga K., Okamoto T. (2018). HIV Tat/P-TEFb Interaction: A Potential Target for Novel Anti-HIV Therapies. Molecules.

[72] Sun Y., Han J., Wang Z., Li X., Sun Y., Hu Z. (2020). Safety and Efficacy of Bromodomain and Extra-Terminal Inhibitors for the Treatment of Hematological Malignancies and Solid Tumors: A Systematic Study of Clinical Trials. Front. Pharmacol..

[73] Huang X.S., Tian R.R., Ma M.D., Luo R.H., Yang L.M., Peng G.H., Zhang M., Dong X.Q., Zheng Y.T. (2022). Bromodomain and Extra-Terminal Inhibitor BMS-986158 Reverses Latent HIV-1 Infection In Vitro and Ex Vivo by Increasing CDK9 Phosphorylation and Recruitment. Pharmaceuticals.

[74] Guo H., Yang W., Li H., Yang J., Huang Y., Tang Y., Wang S., Ni F., Yang W., Yu X.F. (2024). The SAMHD1-MX2 axis restricts HIV-1 infection at postviral DNA synthesis. mBio.

[75] Sharifi H.J., Paine D.N., Fazzari V.A., Tipple A.F., Patterson E., de Noronha C.M.C. (2022). Sulforaphane Reduces SAMHD1 Phosphorylation To Protect Macrophages from HIV-1 Infection. J. Virol..

[76] Dinkova-Kostova A.T., Fahey J.W., Kostov R.V., Kensler T.W. (2017). KEAP1 and Done? Targeting the NRF2 Pathway with Sulforaphane. Trends Food Sci. Technol..

[77] Tokunaga F., Ikeda F. (2022). Linear ubiquitination in immune and neurodegenerative diseases, and beyond. Biochem. Soc. Trans..

[78] Cen X., Li Z., Chen X. (2023). Ubiquitination in the regulation of autophagy. Acta Biochim. Biophys. Sin..

[79] Tian Z., Xu C., He W., Lin Z., Zhang W., Tao K., Ding R., Zhang X., Dou K. (2023). The deubiquitinating enzyme USP19 facilitates hepatocellular carcinoma progression through stabilizing YAP. Cancer Lett..

[80] Liang T., Li G., Lu Y., Hu M., Ma X. (2023). The Involvement of Ubiquitination and SUMOylation in Retroviruses Infection and Latency. Viruses.

[81] Alroy I., Tuvia S., Greener T., Gordon D., Barr H.M., Taglicht D., Mandil-Levin R., Ben-Avraham D., Konforty D., Nir A. (2005). The trans-Golgi network-associated human ubiquitin-protein ligase POSH is essential for HIV type 1 production. Proc. Natl. Acad. Sci. USA.

[82] Nakano Y., Aso H., Soper A., Yamada E., Moriwaki M., Juarez-Fernandez G., Koyanagi Y., Sato K. (2017). A conflict of interest: The evolutionary arms race between mammalian APOBEC3 and lentiviral Vif. Retrovirology.

[83] Azimi F.C., Lee J.E. (2020). Structural perspectives on HIV-1 Vif and APOBEC3 restriction factor interactions. Protein Sci..

[84] Kmiec D., Kirchhoff F. (2024). Antiviral factors and their counteraction by HIV-1: Many uncovered and more to be discovered. J. Mol. Cell Biol..

[85] Hu Y., Knecht K.M., Shen Q., Xiong Y. (2021). Multifaceted HIV-1 Vif interactions with human E3 ubiquitin ligase and APOBEC3s. FEBS J..

[86] Shirakawa K., Takaori-Kondo A., Kobayashi M., Tomonaga M., Izumi T., Fukunaga K., Sasada A., Abudu A., Miyauchi Y., Akari H. (2006). Ubiquitination of APOBEC3 proteins by the Vif-Cullin5-ElonginB-ElonginC complex. Virology.

[87] Andrew A., Strebel K. (2010). HIV-1 Vpu targets cell surface markers CD4 and BST-2 through distinct mechanisms. Mol. Aspects Med..

[88] Sharma S., Jafari M., Bangar A., William K., Guatelli J., Lewinski M.K. (2019). The C-Terminal End of HIV-1 Vpu Has a Clade-Specific Determinant That Antagonizes BST-2 and Facilitates Virion Release. J. Virol..

[89] Song Y.E., Cyburt D., Lucas T.M., Gregory D.A., Lyddon T.D., Johnson M.C. (2018). βTrCP is Required for HIV-1 Vpu Modulation of CD4, GaLV Env, and BST-2/Tetherin. Viruses.

[90] Tokarev A.A., Munguia J., Guatelli J.C. (2011). Serine-threonine ubiquitination mediates downregulation of BST-2/tetherin and relief of restricted virion release by HIV-1 Vpu. J. Virol..

[91] Nodder S.B., Gummuluru S. (2019). Illuminating the Role of Vpr in HIV Infection of Myeloid Cells. Front. Immunol..

[92] Saladino N., Leavitt E., Wong H.T., Ji J.H., Ebrahimi D., Salamango D.J. (2025). HIV-1 Vpr drives epigenetic remodeling to enhance virus transcription and latency reactivation. bioRxiv.

[93] Arora S., Verma S., Banerjea A.C. (2014). HIV-1 Vpr redirects host ubiquitination pathway. J. Virol..

[94] Gu F., Boisjoli M., Naghavi M.H. (2023). HIV-1 promotes ubiquitination of the amyloidogenic C-terminal fragment of APP to support viral replication. Nat. Commun..

[95] Wilkins H.M., Swerdlow R.H. (2017). Amyloid precursor protein processing and bioenergetics. Brain Res. Bull..

[96] Mumby M.J., Prodger J.L., Hackman J., Saraf S., Zhu X., Ferreira R.C., Tomusange S., Jamiru S., Anok A., Kityamuweesi T. (2025). Association between HIV-1 Nef-mediated MHC-I downregulation and the maintenance of the replication-competent latent viral reservoir in individuals with virally suppressed HIV-1 in Uganda: An exploratory cohort study. Lancet Microbe.

[97] Ghaly M., Proulx J., Borgmann K., Park I.W. (2023). Novel role of HIV-1 Nef in regulating the ubiquitination of cellular proteins. Front. Cell. Infect. Microbiol..

[98] Jin J., Zhi X., Wang X., Meng D. (2021). Protein palmitoylation and its pathophysiological relevance. J. Cell. Physiol..

[99] Pei S., Piao H.L. (2024). Exploring Protein S-Palmitoylation: Mechanisms, Detection, and Strategies for Inhibitor Discovery. ACS Chem. Biol..

[100] Zhang M.M., Hang H.C. (2017). Protein S-palmitoylation in cellular differentiation. Biochem. Soc. Trans..

[101] Anwar M.U., van der Goot F.G. (2023). Refining S-acylation: Structure, regulation, dynamics, and therapeutic implications. J. Cell Biol..

[102] Won S.J., Cheung See Kit M., Martin B.R. (2018). Protein depalmitoylases. Crit. Rev. Biochem. Mol. Biol..

[103] Lin D.T.S., Davis N.G., Conibear E. (2017). Targeting the Ras palmitoylation/depalmitoylation cycle in cancer. Biochem. Soc. Trans..

[104] Boncompain G., Herit F., Tessier S., Lescure A., Del Nery E., Gestraud P., Staropoli I., Fukata Y., Fukata M., Brelot A. (2019). Targeting CCR5 trafficking to inhibit HIV-1 infection. Sci. Adv..

[105] Rousso I., Mixon M.B., Chen B.K., Kim P.S. (2000). Palmitoylation of the HIV-1 envelope glycoprotein is critical for viral infectivity. Proc. Natl. Acad. Sci. USA.

[106] Chopard C., Tong P.B.V., Tóth P., Schatz M., Yezid H., Debaisieux S., Mettling C., Gross A., Pugnière M., Tu A. (2018). Cyclophilin A enables specific HIV-1 Tat palmitoylation and accumulation in uninfected cells. Nat. Commun..

[107] Wan J., Liu H., Chu J., Zhang H. (2019). Functions and mechanisms of lysine crotonylation. J. Cell. Mol. Med..

[108] Westerveld M., Besermenji K., Aidukas D., Ostrovitsa N., Petracca R. (2025). Cracking Lysine Crotonylation (Kcr): Enlightening a Promising Post-Translational Modification. ChemBioChem.

[109] Li D., Lin L., Xu F., Feng T., Tao Y., Miao H., Yang F. (2024). Protein crotonylation: Basic research and clinical diseases. Biochem. Biophys. Rep..

[110] Guo Y., Li J., Zhang K. (2024). Crotonylation modification and its role in diseases. Front. Mol. Biosci..

[111] Xie J.Y., Ju J., Zhou P., Chen H., Wang S.C., Wang K., Wang T., Chen X.Z., Chen Y.C., Wang K. (2024). The mechanisms, regulations, and functions of histone lysine crotonylation. Cell Death Discov..

[112] Yang S., Fan X., Yu W. (2024). Regulatory Mechanism of Protein Crotonylation and Its Relationship with Cancer. Cells.

[113] Yan W., Zhang Y., Dai Y., Ge J. (2024). Application of crotonylation modification in pan-vascular diseases. J. Drug Target..

[114] Contreras-de la Rosa P.A., Aragón-Rodríguez C., Ceja-López J.A., García-Arteaga K.F., De-la-Peña C. (2022). Lysine crotonylation: A challenging new player in the epigenetic regulation of plants. J. Proteomics..

[115] Jiang G., Nguyen D., Archin N.M., Yukl S.A., Méndez-Lagares G., Tang Y., Elsheikh M.M., Thompson G.R., Hartigan-O’Connor D.J., Margolis D.M. (2018). HIV latency is reversed by ACSS2-driven histone crotonylation. J. Clin. Investig..

[116] Zhang H., Cai J., Li C., Deng L., Zhu H., Huang T., Zhao J., Zhou J., Deng K., Hong Z. (2023). Wogonin inhibits latent HIV-1 reactivation by downregulating histone crotonylation. Phytomedicine.

[117] Li D., Dewey M.G., Wang L., Falcinelli S.D., Wong L.M., Tang Y., Browne E.P., Chen X., Archin N.M., Margolis D.M. (2022). Crotonylation sensitizes IAPi-induced disruption of latent HIV by enhancing p100 cleavage into p52. iScience.

[118] Zheng Y., Yao X. (2013). Posttranslational modifications of HIV-1 integrase by various cellular proteins during viral replication. Viruses.

[119] Deeks S.G., Lewin S.R., Ross A.L., Ananworanich J., Benkirane M., Cannon P., Chomont N., Douek D., Lifson J.D., Lo Y.R. (2016). International AIDS Society global scientific strategy: Towards an HIV cure 2016. Nat. Med..

[120] Magotra A., Kumar M., Kushwaha M., Awasthi P., Raina C., Gupta A.P., Shah B.A., Gandhi S.G., Chaubey A. (2017). Epigenetic modifier induced enhancement of fumiquinazoline C production in Aspergillus fumigatus (GA-L7): An endophytic fungus from *Grewia asiatica* L.. AMB Express.

[121] Shang H.T., Ding J.W., Yu S.Y., Wu T., Zhang Q.L., Liang F.J. (2015). Progress and challenges in the use of latent HIV-1 reactivating agents. Acta Pharmacol. Sin..

[122] Zhou C.L., Huang Y.F., Li Y.B., Liang T.Z., Zheng T.Y., Chen P., Wu Z.Y., Lai F.Y., Liu S.W., Xi B.M. (2021). A New Small-Molecule Compound, Q308, Silences Latent HIV-1 Provirus by Suppressing Tat- and FACT-Mediated Transcription. Antimicrob. Agents Chemother..

[123] Mediouni S., Lyu S., Schader S.M., Valente S.T. (2022). Forging a Functional Cure for HIV: Transcription Regulators and Inhibitors. Viruses.

[124] Blanco A., Mahajan T., Coronado R.A., Ma K., Demma D.R., Dar R.D. (2021). Synergistic Chromatin-Modifying Treatments Reactivate Latent HIV and Decrease Migration of Multiple Host-Cell Types. Viruses.

[125] Sellmer A., Stangl H., Beyer M., Grünstein E., Leonhardt M., Pongratz H., Eichhorn E., Elz S., Striegl B., Jenei-Lanzl Z. (2018). Marbostat-100 Defines a New Class of Potent and Selective Antiinflammatory and Antirheumatic Histone Deacetylase 6 Inhibitors. J. Med. Chem..

[126] Makhwitine J.P., Kumalo H.M., Ndlovu S.I., Mkhwanazi N.P. (2023). Epigenetic Induction of Secondary Metabolites Production in Endophytic Fungi Penicillium chrysogenum and GC-MS Analysis of Crude Metabolites with Anti-HIV-1 Activity. Microorganisms.

[127] Khoury G., Mota T.M., Li S., Tumpach C., Lee M.Y., Jacobson J., Harty L., Anderson J.L., Lewin S.R., Purcell D.F.J. (2018). HIV latency reversing agents act through Tat post translational modifications. Retrovirology.

[128] Matalon S., Rasmussen T.A., Dinarello C.A. (2011). Histone deacetylase inhibitors for purging HIV-1 from the latent reservoir. Mol. Med..

[129] Rasmussen T.A., Schmeltz Søgaard O., Brinkmann C., Wightman F., Lewin S.R., Melchjorsen J., Dinarello C., Østergaard L., Tolstrup M. (2013). Comparison of HDAC inhibitors in clinical development: Effect on HIV production in latently infected cells and T-cell activation. Hum. Vaccines Immunother..

[130] Wei D.G., Chiang V., Fyne E., Balakrishnan M., Barnes T., Graupe M., Hesselgesser J., Irrinki A., Murry J.P., Stepan G. (2014). Histone deacetylase inhibitor romidepsin induces HIV expression in CD4 T cells from patients on suppressive antiretroviral therapy at concentrations achieved by clinical dosing. PLoS Pathog..

[131] Deeks S.G. (2012). HIV: Shock and kill. Nature.

[132] Boumber Y., Younes A., Garcia-Manero G. (2011). Mocetinostat (MGCD0103): A review of an isotype-specific histone deacetylase inhibitor. Expert. Opin. Investig. Drugs.

[133] Pagans S., Pedal A., North B.J., Kaehlcke K., Marshall B.L., Dorr A., Hetzer-Egger C., Henklein P., Frye R., McBurney M.W. (2005). SIRT1 regulates HIV transcription via Tat deacetylation. PLoS Biol..

[134] Horvath R.M., Brumme Z.L., Sadowski I. (2023). CDK8 inhibitors antagonize HIV-1 reactivation and promote provirus latency in T cells. J. Virol..

[135] Xu H., Wang Y., Lin S., Deng W., Peng D., Cui Q., Xue Y. (2018). PTMD: A Database of Human Disease-associated Post-translational Modifications. Genom. Proteom. Bioinform..

[136] Abner E., Jordan A. (2019). HIV “shock and kill” therapy: In need of revision. Antivir. Res..

[137] Li S., Wang H., Guo N., Su B., Lambotte O., Zhang T. (2023). Targeting the HIV reservoir: Chimeric antigen receptor therapy for HIV cure. Chin. Med. J..

